# The antibiotic resistance and pathogenicity of a multidrug‐resistant *Elizabethkingia anophelis* isolate

**DOI:** 10.1002/mbo3.804

**Published:** 2019-03-19

**Authors:** Mingxi Wang, Hongzhi Gao, Nanfei Lin, Yaping Zhang, Nan Huang, Edward D. Walker, Desong Ming, Shicheng Chen, Shaohua Hu

**Affiliations:** ^1^ Yun Leung Laboratory for Molecular Diagnostics, School of Medicine Huaqiao University Xiamen, Fujian China; ^2^ Clinical Center for Molecular Diagnosis and Therapy Fujian Medical University 2nd Affiliated Hospital Quanzhou, Fujian China; ^3^ Department of Pulmonary and Critical Care Medicine Fujian Medical University 2nd Affiliated Hospital Quanzhou, Fujian China; ^4^ Quanzhou Medical College Quanzhou, Fujian China; ^5^ Department of Microbiology and Molecular Genetics Michigan State University East Lansing Michigan; ^6^ Department of Clinical Laboratory Quanzhou First Hospital Affiliated to Fujian Medical University Fujian China; ^7^ State Key Laboratory for Diagnosis and Treatment of Infectious Diseases, Collaborative Innovation Center for Diagnosis and Treatment of Infectious Diseases The First Affiliated Hospital of Medical College, Zhejiang University Hangzhou, Zhejiang China

**Keywords:** antibiotic resistance mechanisms, comparative genomic analysis, *Elizabethkingia anophelis*, genome sequencing, pathogenicity mechanisms

## Abstract

*Elizabethkingia anophelis* 12012‐2 PRCM was isolated from a patient with multiple organ dysfunction syndrome and lower respiratory tract infection in China. Minimum inhibitory concentration (MIC) analysis demonstrated that it was resistant to 20 antibiotics including trimethoprim/sulfamethoxazole and ciprofloxacin, which were effective for the elimination of other *Elizabethkingia* infections. To investigate multidrug resistance and pathogenicity mechanisms, we analyzed genome features of 12012‐2 PRCM and compared them to the other *Elizabethkingia* species. The draft genome size was 4.02 Mb with a GC content of 32%, comparable to that of other *E. anophelis* strains. Phylogenetic analysis showed that *E. anophelis* 12012‐2 PRCM formed a sister group with *E. anophelis* 502, distinct from clades formed by other clinical and environmental *E. anophelis* isolates. *E. anophelis* 12012‐2 PRCM contained multiple copies of *β*‐lactamase genes as well as genes predicted to function in antimicrobial efflux. It also contained 92 genes that were potentially involved in virulence, disease, and defense, and were associated with resistance and pathogenicity. Comparative genomic analysis showed high homology among three clinical and two environmental *E. anophelis* strains having a variety of similar antibiotic resistance and virulence factor genes, and similar genomic structure. Applications of this analysis will contribute to understanding the antibiotic resistance and pathogenic mechanisms of *E. anophelis *infections, which will assist in the management of infections as it increases in prevalence.

## Graphical abstract

To investigate the possible multidrug resistance and pathogenicity mechanisms, we investigated 12012‐2 PRCM genome features and compared them to the other *Elizabethkingia* species. Comparative genomic analysis showed high homology among three clinical and two environmental *E. anophelis* stains that contained a variety of similar antibiotic resistance genes and VF genes, and similar genomic structure. Applications of this analysis will contribute to understanding the antibiotic resistance and pathogenic mechanisms of *E. anophelis* infections, which will assist in the management of this bacterium as it increases in prevalence.

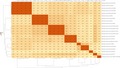

## 1 INTRODUCTION

*Elizabethkingia anopheles *(*E. anophelis*) is an aerobic, nonmotile, gram‐negative, rod‐shaped bacterium (Kampfer et al., 2011). It is an emerging, opportunistic, nosocomial pathogen (Frank et al., 2013; Lau et al., 2016, 2015; Teo et al., 2013). Neonates (Frank et al., 2013; Lau et al., 2016, 2015), postsurgery patients (Teo et al., 2013), or old people with underlying diseases (Lau et al., 2016) are most susceptible to *E. anophelis* infections. It has caused infections and outbreaks in Singapore, Hong Kong, and the United States (Frank et al., 2013; Janda & Lopez, 2017; Lau et al., 2015; Teo et al., 2013). The largest outbreak in the United States (65 cases) was recorded in hospitalized, immune‐compromised patients in the Great Lakes region, including Wisconsin, Michigan, and Illinois, with a high mortality rate (20/65, 30.8%) (https://www.cdc.gov/elizabethkingia/outbreaks/).

The unknown pathogenesis mechanisms, multidrug resistance mechanisms, and misclassifications as other bacteria complicate management of *E. anophelis* infections (Frank et al., 2013; Hu, Jiang, Zhang et al., 2017; Lau et al., 2016, 2015). Routine phenotypic and biochemical tests often fail to distinguish them from other bacteria; moreover, *E. anophelis* has been frequently misdiagnosed as *E. meningoseptica* (previously known as *Chryseobacterium meningosepticum*) with automated microbial identification systems (Kampfer et al., 2011; Lau et al., 2016, 2015; Nicholson et al., 2016; Teo et al., 2013). Often, molecular methods (i.e., the 16SrRNA sequencing, MALDI‐TOF MS) fail to resolve different *Elizabethkingia* species (Breurec et al., 2016; Han et al., 2017). Empirical treatments are difficult because of multiple drug resistance and lack of drug susceptibility testing standards for these bacteria. Particularly, our knowledge of the antibiotic resistance spectra and the resistance mechanisms remain limited in *E. anophelis* because it is a relatively newly discovered bacterium. The pathogenesis mechanisms in *Elizabethkingia *remain unclear. Strains isolated during the outbreak in Wisconsin harbored a mutation in the *MutY* gene which is involved in DNA repair (Perrin et al., 2017), but the relevance of it to virulence is unknown.

Our *E. anophelis* strain, 12012‐2 PRCM, was isolated from a patient with multiple organ dysfunction syndrome (MODS) (Hu, Jiang, Zhang et al., 2017). This isolate was not susceptible to any selected antibiotics, demonstrating it was a multidrug‐resistant strain. Therefore, the aim of this study was to investigate drug resistance and pathogenesis mechanisms. We performed genome sequencing for *E. anophelis *12012‐2PRCM and conducted a comparative genomic analysis to those in other environmental and clinical isolates. Our results contribute to the management of *Elizabethkingia* infection and the better understanding the pathogenicity of *E. anophelis*.

## 2 MATERIALS AND METHODS

### 2.1 DNA extraction and antimicrobial susceptibility testing

A multidrug‐resistant *E. anophelis* strain, designated 12012‐2PRCM, was isolated from an 82‐year‐old male patient presenting with MODS and lower respiratory tract infection (Hu, Jiang, Zhang et al., 2017). Antimicrobial susceptibility testing (AST), bacteria culturing, and genomic DNA extraction were done as previously performed (Hu, Jiang, Zhou et al., 2017).

### 2.2 Whole‐genome sequencing, assembly, and annotation for *E. anophelis* 12012‐2PRCM

Genome sequencing was done with the MiSeq instrument (Illumina, Inc., San Diego, CA) using 500 bp library preparations. Raw data processing and genome assembly were performed by the SOAPdenovo 2.04‐r240 version (Li et al., 2010). After assembly, we obtained a 402,331,983‐bp genome containing 83 contigs and 76 scaffolds. It was deposited into GenBank (LPXG00000000). The genome annotation was done with RAST (Aziz et al., 2008; Overbeek et al., 2014).

### 2.3 Comparative genomic analysis of the *E. anophelis* isolates

The whole‐genome phylogenetic tree of 22 *Elizabethkingia* species was constructed using REALPHY (Reference sequence Alignment‐based Phylogeny builder) with default parameters (Bertels, Silander, Pachkov, Rainey, & Nimwegen, 2014). It included 14 clinically pathogenic strains, four human‐associated strains, and four environmental isolates (Table 1).

**Table 1:** General genomic characteristics of 22 *Elizabethkingia anophelis* strains

Sources	Strain	Site of isolation	Type	Assembly No.	Level	Scaffold	Size (Mb)	GC (%)	Protein	rRNA	tRNA	Other RNA	Gene	Pseudo gene
	**12012‐2 PRCM**	**sputum**	**—**	**NZ_LPXG00000000.1**	**—**	**83**	**4.02**	**35.6**	**3,554**	**—**	**42**	**1**	**3,680**	**82**
Clinically pathogenic *E. anophelis*	NUHP1	cardiothoracic	Chr	CP007547.1	complete	1	4.37	35.6	3,912	15	51	3	4,039	58
	FMS‐007	sputum	Chr	CP006576.1	complete	1	3.94	35.6	3,480	15	52	3	3,593	43
	CSID_3015183684	Blood	Chr	CP015066.2	complete	1	3.93	35.8	3,472	15	52	3	3,579	37
	0422	blood	Chr	CP016370.1	complete	1	3.99	35.6	3,564	15	50	3	3,679	47
	F3543	CSF	Chr	CP014340.1	complete	1	3.97	35.6	3,512	15	52	3	3,632	50
	FDAARGOS_198	blood	Chr	CP023010.1	complete	1	4.07	35.8	3,529	15	52	3	3,738	139
	502	wound swab	**—**	NZ_AVCQ00000000.1	**—**	21	3.96	35.5	3,676	12	43	**—**	3,731	**—**
	NUHP2	cardiothoracic	**—**	NZ_ASYF00000000.1	**—**	59	4.33	35.5	3,891	N/A	42	3	4,025	86
	NUHP3	cardiothoracic	**—**	NZ_ASYG00000000.1	**—**	71	4.33	35.5	3,883	N/A	43	3	4,031	99
	NUH1	hygiene sink aerator of the cardiothoracic surgery suite	**—**	NZ_ASYH00000000.1	**—**	59	4.33	35.5	3,895	N/A	44	3	4,031	86
	NUH4	hand hygiene sink aerator of the surgical stepdown	**—**	NZ_ASYI00000000.1	**—**	50	4.24	35.6	3,815	N/A	44	3	3,949	84
	NUH6		**—**	NZ_ASYJ00000000.1	**—**	74	4.12	35.6	3,712	N/A	44	3	3,848	86
	NUH11	hand hygiene sink aerator of the neonatal ICU	**—**	NZ_ASYK00000000.1	**—**	59	4.09	35.6	3,651	4	45	3	3,792	89
Environmental *E. anophelis*	Ag1	*A*N*opheles gambiae*	Chr	CP023402.1	complete	1	4.09	35.5	3,676	15	52	3	3,780	34
	R26	*A*N*opheles gambiae* G3 adults	Chr	CP023401.1	complete	1	4.06	35.5	3,634	15	52	3	3,737	33
	AR4‐6	*A*N*opheles si*N*e*N*sis*	Chr	CP023404.1	complete	1	4.09	35.5	3,676	15	52	3	3,780	34
	AR6‐8		Chr	CP023403.1	complete	1	4.09	35.5	3,676	15	52	3	3,780	34
Human‐associated *E. anophelis*	CSID_3015183678	N/A	Chr	CP014805.2	complete	1	3.93	35.8	3,473	15	52	3	3,578	35
	CSID_3000521207	N/A	Chr	CP015067.2	complete	1	3.85	35.7	3,400	15	52	3	3,505	35
	CSID_3015183681	N/A	Chr	CP015068.2	complete	1	3.93	35.8	3,471	15	52	3	3,578	37
	3375	N/A	Chr	CP016373.1	complete	1	4.01	35.7	3,578	15	54	3	3,704	54

Note

Chr: chromosome; N/A: not available, Bold values: We isolated and sequenced

The average nucleotide identity (ANI), pan‐genome, and core genome were analyzed by EDGAR 2.0 (Blom et al., 2016). The CRISPs (Clustered Regularly Interspaced Short Palindromic repeat sequences) were predicted by CRISPR recognition tool (CRT) (Bland et al., 2007). ICEberg database was used to detect for integrative and conjugative elements (ICE)(Bi et al., 2012).The resistance genes and VFs were searched (BLASTp) against the CARD database (Jia et al., 2017; McArthur et al., 2013; McArthur & Wright, 2015) and the VFDB protein Set B database (Chen, Xiong, Sun, Yang, & Jin, 2012; Chen, Zheng, Liu, Yang, & Jin, 2016), respectively, by collaborating with Beijing Novogene Bioinformatics Technology Co., Ltd. (BNNT), followed by filtering with more stringent cutoff parameters as described previously (Hu et al., 2018) and two additional cutoff parameters, Match length >100 amino acids and Identical >100 amino acids.

Alignment of five *E. anophelis* genomes, including the strain described here, was completed with Progressive Mauve (Darling, Mau, & Perna, 2010). The genomic data of the four other strains were downloaded from the GenBank database. *E. anophelis *NUHP1 (CP007547) was isolated in 2012 from a patient in the cardiothoracic ICU ward of National University Hospital, Singapore. *E. anophelis* CSID3000521207 (CP015067) was isolated in 2016 from a patient in Wisconsin, USA. *E. anophelis *Ag1 (AHHG00000000) was isolated in 2010 from the gut of an *Anophelis gambiae* mosquito in a laboratory colony in New Mexico, USA. *E. anophelis *R26 (MAHN00000000) was isolated in 2006 from Anophelis gambiae G3 adults in a laboratory colony in Sweden. The latter two environmental strains (Ag1, R26) had been used as reference stains to analyze the genes of antibiotic resistance and VFs in the hospital isolated *E. anophelis* strains (Teo et al., 2014).

## 3 RESULTS AND DISCUSSION

### 3.1 Genomic features of *E. anophelis* 12012‐2PRCM

**Figure 1:**
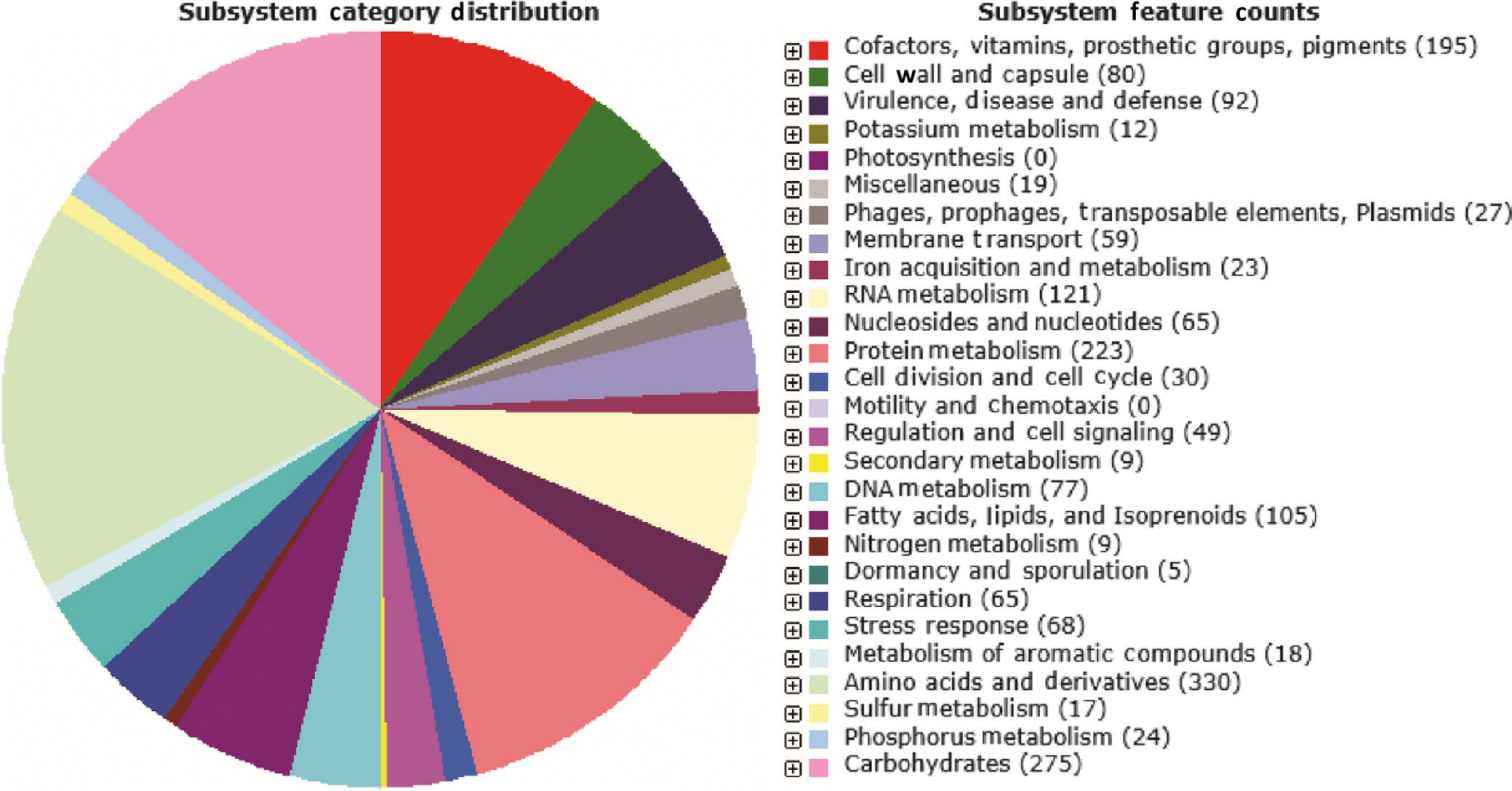
Subsystem distribution predicted from the genome of *Elizabethkingia anophelis* 12012‐2PRCM strain. Each portion of the circular graph displays different function classification and percentages of the gene numbers in the same function classification. The number in parentheses is the gene number within the same function classification

The assembly of strain 12012‐2PRCM sequence data generated 83 scaffolds. It had a genome of 4.02 M bp with an average GC content of 35.5%. *E. anophelis* 12012‐2PRCM had 3,680 genes including 3,554 protein‐encoding genes, 82 pseudogenes, and 42 tRNAs (Table 1). The RAST showed that *E. anophelis *12012‐2PRCM genome had 27 subsystems that consisted of 87 categories (Figure 1). At least 330, 275, 268, and 121 CDSs were assigned to the “amino acid and derivatives,” “carbohydrate metabolism,” “protein metabolism,” and “RNA metabolism” categories, respectively. Moreover, the “virulence, disease and defense” category contained 92 CDSs that were involved in resistance to antibiotics and toxic compounds, indicating that this strain was possibly resistant to multiple antibiotics (also see below).

### 3.2 Phylogenetic inferences

**Figure 2:**
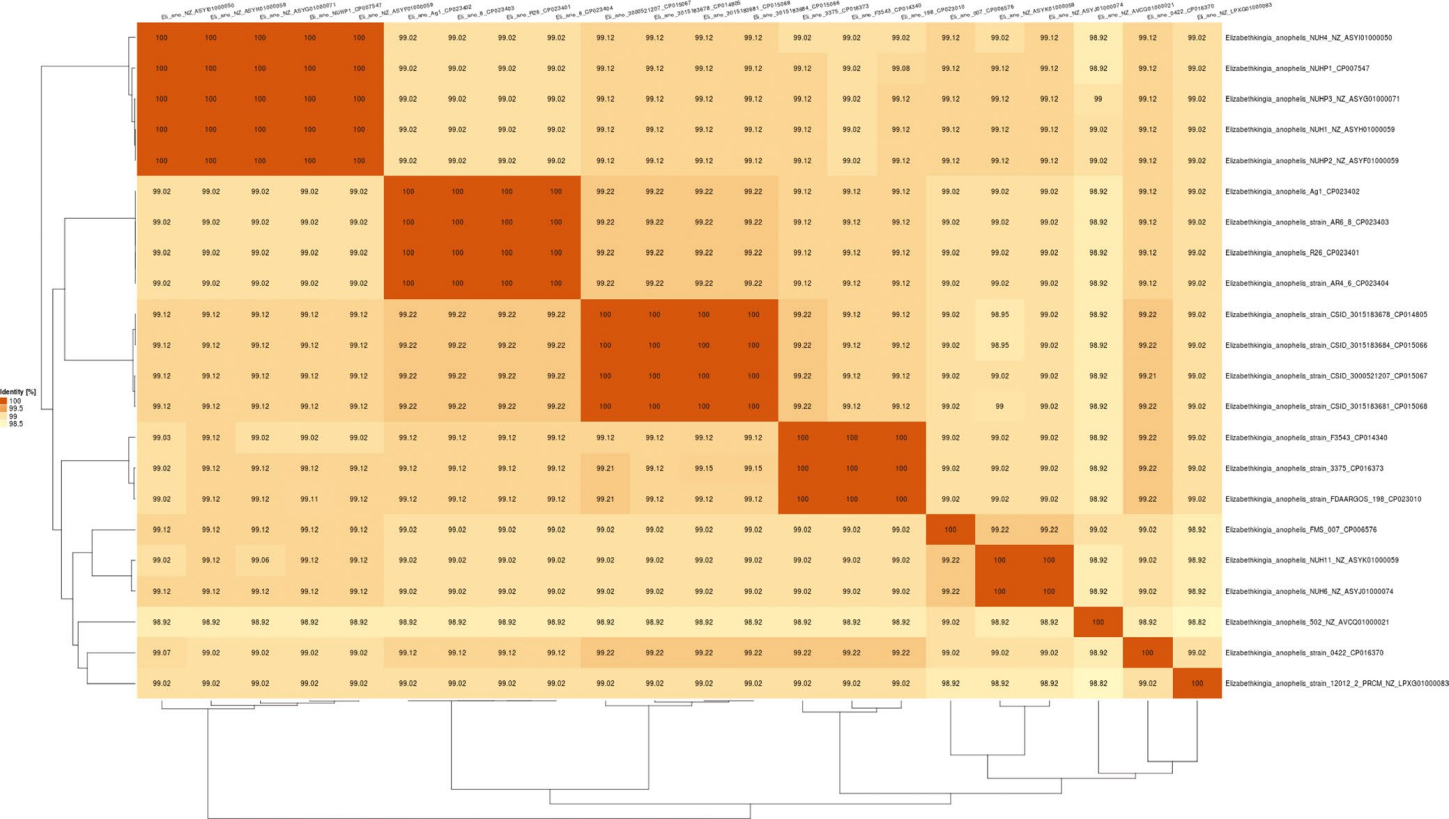
Heat map of ANI values among representative *Elizabethkingia anophelis* species

**Figure 3:**
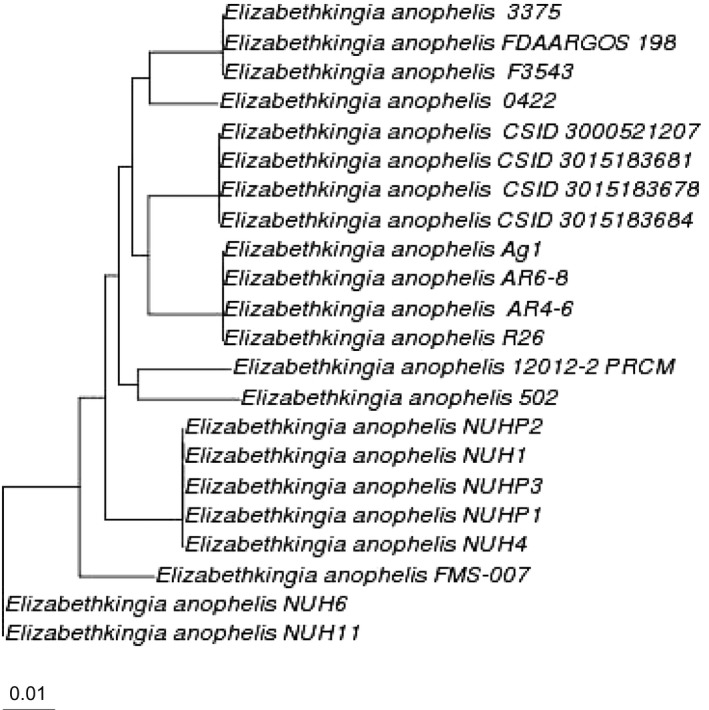
Whole‐genome phylogenetic tree of 22 *Elizabethkingia anophelis* species. This tree was created through REALPHY with the default parameters

12012‐2 PRCM showed a high ANI (>99%) with the typical species *E. anophelis* R26, and ANI (>98%) with all other selected *E. anophelis* strains (Figure 2), indicating that it is a strain of *E. anophelis*. The phylogenetic tree demonstrated that *E. anophelis *12012‐2 PRCM was clustered together with *E. anophelis* 502 that was isolated from a patient with a trauma wound in the United Kingdom (Figure 3). These two strains formed a separate group which departed from other clinical‐ or mosquito‐associated isolates, indicating that they evolved following the different pathways. It is worth highlighting that Wisconsin outbreak isolates (*E. anophelis *CSID 3000521207, CSID 3015183678, CSID 3015183681, and CSID 3015183684) formed an independent clade from isolates from Singapore (e.g., NUHP2, NUH1, NUHP1, NUPH3, and NUH3), suggesting that they may originate from different sublines.

**Figure 4:**
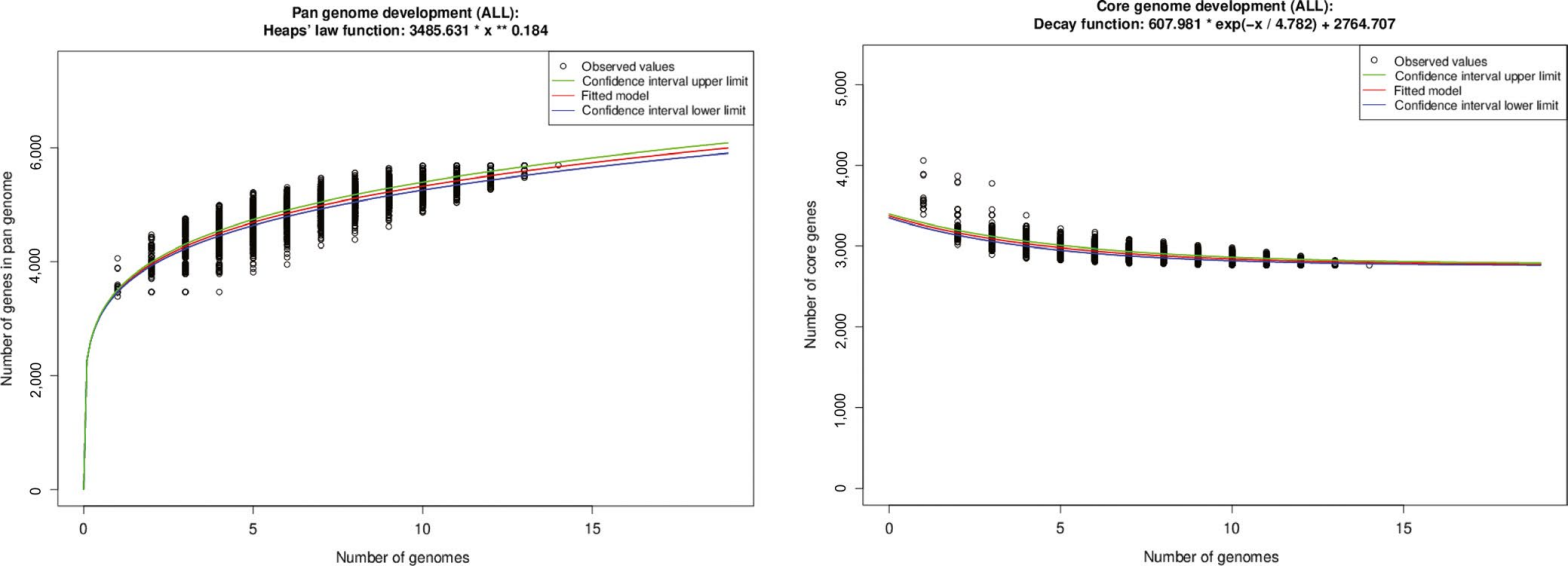
Core and pan genome evolution according to *Elizabethkingia anophelis* strain. Right: Total number of genes (pan genome) for a given number of genomes sequentially added. Left: Number of ubiquitous genes (core genome) as a function of the number of genomes sequentially added

The predicted protein sequences were used for core and pan‐genome development analysis among the selected 15 *E. anophelis* genomes. *E. anophelis* displayed an open pan‐genome because the total number of genes in pan‐genomes increased with the increasing input genome. Also, the number of core genes decreased with the increasing input genomes. A total of 4.8 new genes/added genome were expected using the formula derived from the singleton development plot (Figure 4). The core genome for the 15 selected *E. anophelis* was calculated to be 2,764 CDS per genome.

### 3.3 Antibiotic susceptibility profiles of *E. anophelis* 12012‐2PRCM

The antimicrobial susceptibility of *E. anophelis* remains unclear. *E. anophelis* 12012‐2PRCM was highly resistant to 20 antibiotics in our drug susceptibility test, indicating that it was a multidrug‐resistant strain. These drugs belong to seven classes including aminoglycosides, *β*‐lactams, polypeptides, sulfonamides, chloramphenicols, quinolones, and tetracyclines (Table A1).

Resistance to tetracycline, trimethoprim/sulfamethoxazole, and ciprofloxacin raised a serious concern because these drugs have been widely used for treatment of infections of *Elizabethkingia* species. For example, all 51 *E. anophelis* isolates from South Korea were immediately sensitive or sensitive to piperacillin or piperacillin–tazobactam (Han et al., 2017). Furthermore, 25 Wisconsin outbreak strains were also susceptible to piperacillin–tazobactam tested by Kirby Bauer disk diffusion method (Perrin et al., 2017). The same observations were reported in *E. anophelis* EM361‐97 isolated from Taiwan (Lin, Lai, Yang, Huang, & Lin, 2017). Our isolate was resistant to piperacillin and piperacillin–tazobactam, indicating that 12012‐2PRCM had different antibiotic resistance mechanisms from the above strains. However, the antibiograms in various *Elizabethkingia* isolates are often controversially reported. For instance, most of the 100 *E. anophelis* strains isolated from Korea as well as strain EM361‐97 from Taiwan were resistant to ciprofloxacin and levofloxacin, while most of the Wisconsin outbreak strains were susceptible to these quinolone drugs (Han et al., 2017; Lin et al., 2017; Perrin et al., 2017). These variations stress that different origins of *Elizabethkingia* isolates may evolve different antibiotic resistance mechanisms. However, it should be noted that the clinical significance of the above differences remains unknown due to the lack of interpretative breakpoints for antimicrobial resistance in *E. anophelis*.

### 3.4 Resistome analysis

Antibiotic resistance genes were predicted by searching the CARD database (Jia et al., 2017; McArthur et al., 2013). At least eight classes of antibiotic resistance genes were found in *E. anophelis* 12012‐2 PRCM (Table 2).

**Table 2:** The predicted antibiotic resistance genes in five *E. anophelis *isolates: 12012‐2PRCM, CSID3000521207, NUHP1, Ag1, R26

Category	12012‐2 PRCM	CSID 3000521207	NUHP1	Ag1	R 26
Efflux pump complex or subunit conferring antibiotic resistance	*qacH*	*qacH*	*qacH*	*qacH*	*qacH*
	*abeS*	*abeS*	*abeS*	*abeS*	*abeS*
Determinant of elfamycin resistance	*LpxC*	*LpxC*	*LpxC*	*LpxC*	*LpxC*
	*SPM‐1*	*SPM‐1*	*SPM‐1*	*SPM‐1*	*SPM‐1*
Determinant of phenicol resistance	*catB2*	*catB2*	*catB2*	*catB2*	*catB2*
	*catB6*	*catB6*	*catB6*	*catB6*	*catB6*
	*catB7*	*catB7*	*catB7*	*catB7*	*catB7*
	*catB8*	*catB8*	*catB8*	*catB8*	*catB8*
	*catB9*	*catB9*	*catB9*	*catB9*	*catB9*
	*catB10*	*catB10*	*catB10*	*catB10*	*catB10*
Antibiotic inactivation enzyme	*tetX*	*—*	*tetX*	*tetX*	*tetX*
	*catB3*	*catB3*	*catB3*	*catB3*	*catB3*
	*LRA‐19*	*LRA‐19*	*—*	*LRA‐19*	*LRA‐19*
	*PEDO‐2*	*PEDO‐2*	*—*	*PEDO‐2*	*PEDO‐2*
	*LRA‐12*	*LRA‐12*	*—*	*LRA‐12*	*LRA‐12*
	*PEDO‐3*	*PEDO‐3*	*PEDO‐3*	*PEDO‐3*	*PEDO‐3*
	*—*	*TLA‐1*	*TLA‐1*	*—*	*—*
	*—*	*TLA‐3*	*TLA‐3*	*—*	*—*
	*arr‐1*	*—*	*—*	*—*	*—*
Determinant of fluoroquinolone resistance	*rpsJ*	*rpsJ*	*rpsJ*	*rpsJ*	*rpsJ*
	*tetB(48)*	*tetB(48)*	*tetB(48)*	*tetB(48)*	*tetB(48)*
Determinant of β‐lactam resistance	*CPS‐1*	*CPS‐1*	*CPS‐1*	*CPS‐1*	*CPS‐1*
	*ESP‐1*	*ESP‐1*	*ESP‐1*	*ESP‐1*	*ESP‐1*
	*PEDO‐1*	*PEDO‐1*	*PEDO‐1*	*PEDO‐1*	*PEDO‐1*
	*LRA‐17*	*LRA‐17*	*—*	*LRA‐17*	*LRA‐17*
	*—*	*—*	*LRA‐12*	*—*	*—*
	*—*	*—*	*PEDO‐2*	*—*	*—*
	*—*	*TEM‐113*	*TEM‐113*	*TEM‐113*	*TEM‐113*
Determinant of streptogramin resistance	*ErmF*	*—*	*—*	*—*	*—*
	*Erm(35)*	*—*	*—*	*—*	*—*
Determinant of diaminopyrimidine resistance	*dfrE*	*dfrE*	*dfrE*	*dfrE*	*dfrE*

Note

—: not predicted

*Elizabethkingia *bacteria are well known to be highly resistant to *β*‐lactam drugs as shown in this study and others. Piperacillin, an expanded‐spectrum penicillin, can be hydrolyzed by several *β*‐lactamases. *E. anophelis* 12012‐2 PRCM carried at least four *β*‐lactamase genes (*CPS‐1*, *ESP‐1*, *PEDO‐1, *and *LRA‐17*). *CPS‐1* encoding a subclass of B3 metal‐beta‐lactamase was first isolated from *Chryseobacterium piscium*. It conferred resistance to penicillin, cephalosporin, carbapenem as well as other *β*‐lactams (Gudeta et al., 2015). The products of *CPS‐1* and *PEDO‐1* (encoding another subclass B3 metal‐beta‐lactamase) significantly increased the MICs of ampicillin, ceftazidime, cefpodoxime, cefoxitin, and meropenem (Gudeta et al., 2016). The clinically relevance of *β*‐lactamase LRA‐17 remains unclear, but the presence of this novel *β*‐lactamase of environmental origin could contribute to the resistance spectrum of these bacteria (Allen, Moe, Rodbumrer, Gaarder, & Handelsman, 2009).

The resistance to the fluoroquinolones ciprofloxacin and levofloxacin can be explained by the mutational DNA gyrase A subunit (gyrA). For *Elizabethkingia*, two mutations (Ser83Ile and Ala709Ser) were found in the gyrA protein (Lin, Lai, Yang, Huang, & Lin, 2018). Ser83Ile possibly leads to the increased MICs to ciprofloxacin and levofloxacin in strain 12012‐2PRCM as shown in a recent study. However, the effects of the second mutation (Ala709Ser) at C‐terminal of gyrA on the fluoroquinolone resistance have not been documented in *Elizabethkingia*. Besides the mutational *gyrA*, the fluoroquinolone‐resistant genes, *rpsJ* *and tetB(48)*, were discovered in strain 12012‐2PRCM, which may also contribute to the resistance to fluoroquinolones.

Elizabethkingia *anophelis* 12012‐2 PRCM carried the factor TetX, shown in *E. coli *to efficiently degrade tetracycline (Yang et al., 2004). All five *E. anophelis* strains contained many *catB* genes or cat variants (Table 2), which usually play a role in the composition of gene cassette or integron, and confer to the ability of antibiotic resistance. The resistance action mechanisms of* catB* were already clarified in our previous report (Hu et al. 2018). Genes such as *LpxC *and *SPM‐1*, *ErmF, *and* Erm(35) *as well as *dfrE *conferred resistance to diaminopyrimidine, streptogramin, and elfamycin, respectively. Elizabethkingia *anophelis* 12012‐2 PRCM also contained nine genes encoding antibiotic inactivation enzymes.

### 3.5 Comparative analysis of the virulence factor genes in *E. anophelis* strains

The homologs of the virulence factors (VFs) in *E. anophelis *isolates were investigated using the VFDB Set B database (Chen et al., 2012, 2016). Up to 25, 28, 26, and 26 VFs were identified in strains 12012‐2PRCM, CSID3000521207, Ag1, and R26, respectively (Table 3). These VFs involved in the capsule formation, lipopolysaccharide or lipid biosynthesis and metabolism, ion transport protein, stress response (heat shock protein, catalase, peroxidase, superoxide dismutase), secretion system, and several others. Compared to Wisconsin strain CSID3000521207, some variations were found in these VFs in 12012‐2PRCM. For example, the genes *fcl*, *dfoC, dfoJ*, *rmlC, bplG, *and *gmd *were absent in 12012‐2PRCM. However, CSID3000521207 lacked virulence genes *capL *and *pglC*.

**Table 3:** The predicted virulence factor genes in 12012‐2PRCM, CSID3000521207, Ag1, and R26

VF Classification	Genes coding for virulence factors				Encoded VF proteins
	Clinically pathogenic *E. anophelis*		Environmental *E. anophelis*		
	12012‐2 PRCM	CSID 3000521207	Ag1	R26	
Capsule	***capL***	***—***	***capL***	***capL***	Hypothetical protein
	*+*	*+*	*+*	*+*	M3Q_285 Nucleoside‐diphosphate sugar epimerase
	*ugd*	*ugd*	*ugd*	*ugd*	UDP‐glucose 6‐dehydrogenase
Capsule biosynthesis and transport	*—*	*fcl*	*—*	*—*	GDP‐fucose synthetase
Catalase	***katA***	***katA***	***katA***	***katA***	Catalase
Catalase‐peroxidase	*katG*	*katG*	*katG*	*katG*	Catalase
ClpP	***clpP***	***clpP***	***clpP***	***clpP***	ATP‐dependent Clp protease proteolytic subunit
Desferrioxamine	*dfoA*	*dfoA*	*dfoA*	*dfoA*	L‐lysine 6‐monooxygenase involved in desferrioxamine biosynthesis
	*—*	*dfoC*	*dfoC*	*dfoC*	Desferrioxamine siderophore biosynthesis protein dfoC
	*—*	*dfoJ*	*—*	*—*	Putative decarboxylase involved in desferrioxamine biosynthesis
EF‐Tu	***tuf***	***tuf***	***tuf***	***tuf***	Translation elongation factor Tu
Exopolysaccharide	*pgi*	*pgi*	*pgi*	*pgi*	Glucose‐6‐phosphate isomerase
GPL locus	*rmlA*	*rmlA*	*rmlA*	*rmlA*	RmlA
Heme biosynthesis	*hemL*	*hemL*	*hemL*	*hemL*	Glutamate‐1‐semialdehyde aminotransferase
Hsp60	***htpB***	***htpB***	***htpB***	***htpB***	60‐kDa chaperonin protein, Cpn60groEL protein Heat shock protein B
IlpA	*IlpA*	*IlpA*	*IlpA*	*IlpA*	Immunogenic lipoprotein A
Isocitrate lyase	*icl*	*icl*	*icl*	*icl*	Isocitrate lyase
LOS	*−*	*+*	*+*	*+*	C8J_1084 Hypothetical protein
	*galE*	*galE*	*galE*	*galE*	UDP‐glucose 4‐epimerase
LPS	*—*	*bplG*	*—*	*—*	Probable sugar transferase
	*—*	*gmd*	*—*	*—*	GDP‐mannose 4,6‐dehydratase
Methionine sulphoxide reductase	*msrA/BpilB*	*msrA/BpilB*	*msrA/BpilB*	*msrA/BpilB*	Peptide methionine sulfoxide reductase
Mg2+ transport	*mgtB*	*mgtB*	*mgtB*	*mgtB*	Hypothetical protein
MOMP	***DnaK***	***DnaK***	***DnaK***	***DnaK***	Molecular chaperone
N‐linked protein glycosylation	*pglC*	*—*	*pglC*	*pglC*	Putative galactosyltransferase
Polar flagella	*flmH*	*flmH*	*flmH*	*flmH*	3‐oxoacyl‐ACP reductase
Streptococcal enolase	***eno***	***eno***	***eno***	***eno***	Phosphopyruvate hydratase
T4SS effectors	*+*	*+*	*+*	*+*	COXBURSA331_A0369 Trans‐2‐enoyl‐CoA reductase (no unique name)

Note

+: presence;—: absence; bold: were discussed in Hu et al. 2018; underlined: consistent to the virulence factors in R26, Ag1 predicted by Breurec et al. (Breurec et al., 2016).

Strain 12012‐2PRCM may be a truly emerging pathogen due to these conserved VFs widely identified in *Elizabethkingia* other pathogens. For example, *katG* encoding a bacterial catalase‐peroxidase (heme enzyme) was found to be involved in the iron metabolism and stress response. Beside the iron metabolism, KatG activated the prodrug isoniazid, which was involved in *Mycobacterium tuberculosis* pathogenesis course (Pym et al., 2001). IlpA, a membrane‐bound lipoprotein, has been known to function as an adhesion factor in *Vibrio vulnificus*. It helps the adhesion to human immune cells through its C‐terminal domain. Consequentially, it induces cytokine production, which plays an important role in *V. vulnificus* infection (Goo, Han, Kim, Lee, & Park, 2007; Lee et al., 2010). One can assume the same physiological roles in 12012‐2‐PRCM due to their good amino acid sequence homology. The presence of* IlpA *in our strain 12012‐2 PRCM implied that it might also have the potential to cause septicemia. Other virulence factor genes such as *clpP*, *tuf*, r*mlA*, *htpB,* and *DnaK* may be involved in defense or invasion during the course of pathogenesis, already discussed in our previous report (Hu et al. 2018). In addition, it is worth noting that *E. anophelis* isolates from mosquitoes also shared these conserved virulence factors. However, their potential for pathogenicity in humans have not been investigated.

### 3.6 Prophages and conjugative transposons in the selected *Elizabethkingia*

**Figure A1:**
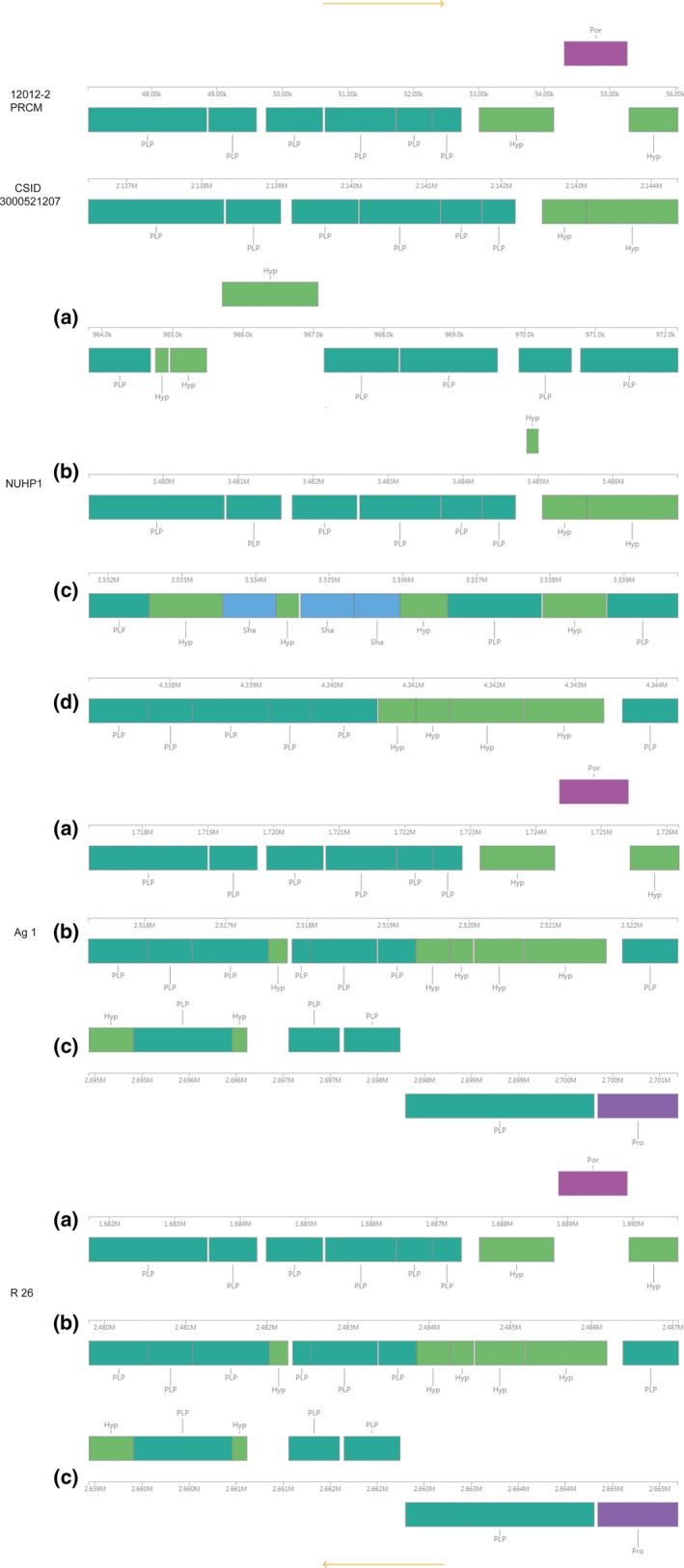
Prophage regions with predicted elements in selected *Elizabethkingia anophelis *strains. Different colored rectangles meant different phage elements. Pro: protease; Plp: phage‐like protein; Hyp: hypotheical protein; Sha: tail shaft; Pro: portal protein

All five *E. anophelis* genomes contained incomplete prophage (Figure A1). In our strain 12012‐2 PRCM, only one prophage was identified. It had nine CDs located at 47,038 bp‐56041 bp (9 kb). The strain CSID 3000521207 also contained one 7.8‐kb prophage extending from 2,136,491 bp to 2,144,356 bp. NUHP1 was predicted to carry four prophages (8.3 kb, 7.8 kb, 7.9 kb, and 7.2 kb, respectively) (Figure A1). Strains Ag1 and R26 shared three prophages (8.9 kb, 7.2 kb, and 6.2 kb, respectively), although the prophages were located on different sites in two of the genomes (Figure A1), implying that genome rearrangements existed. Of interest, our strain 12012‐2 PRCM shared one prophage of Ag1 and R26 while prophage of CSID 3000521207 was similar to the one in NUHP1 (Figure A1), demonstrating that prophages in *E. anophelis* species were conserved. However, among these predicted prophages, many elements were lost. For example, a significant component integrase (a marker for mobile DNA elements and participating in bacteria pathopoiesis (Liu et al., 2015) was not predicted in any of the above prophages.

**Figure A2:**
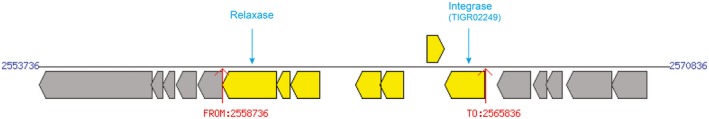
Structure of ICE identified in 12012–2 PRCM genome.Rectangles indicate different ORFs. The yellow colorshighlightIntegrative and Conjugative Elements. The location of ICE ranged from 2,558,736 bp to 2,565,836 bp

Horizontal gene transfer (HGT) plays a huge role in microbial evolution, allowing microbes to acquire new genes and phenotypes (Banuelos‐Vazquez, Torres Tejerizo, & Brom, 2017). Integrative and conjugative elements (ICEs), also called conjugative transposons, are a diverse group of mobile genetic elements found in both gram‐positive and gram‐negative bacteria (Johnson & Grossman, 2015; Wozniak & Waldor, 2010). ICEs use a range of mechanisms to promote their core functions of integration, excision, transfer, and regulation, contributing to bacterial pathogenesis (Banuelos‐Vazquez et al., 2017; Johnson & Grossman, 2015; Wozniak & Waldor, 2010). In our strain 12012–2 PRCM, using the database ICEberg 2.0, a putative ICE region (location: 2,558,736 to 2,565,836 bp) was identified. In this mobile genetic element, both relaxase and integrase (TIGR02249) were predicted (Figure A2). The CSID 3000521207, one present representative isolate of the outbreak in Wisconsin, had the integrative and conjugative element ICEEa1 (Perrin et al., 2017). ICEEa1 consists of VirD4 ATPase (T4CP), relaxase, integrase, and several Tra proteins. This transposon element inserted into and disrupted the gene *MutY* (an adenine DNA glycosylase that is required for fixing G‐A mis‐pairs), making the strain more liable to mutation and outbreak infection (Perrin et al., 2017). Recent research showed that ICEs were ubiquitous in *E. anophelis* species; 31 of selected 36 *E. anophelis* strains (86%) harbored 32 ICEs (Xu, Pei, Nicholson, Lan, & Xia, 2018). These ICEs were classified into three types: ICEEaI, ICEEaII, and ICEEaIII. For example, conjugative elements ICEEaII and ICEEaIII were identified in the Singapore outbreak strain NUHP1. Also, the Anopheles mosquito strains Ag1 and R26 contained ICEEaIII (Xu et al., 2018). More detailed analysis of ICEs will clarify pathogenesis and drug resistance mechanisms of *E. anophelis*.

### 3.7 Synteny analysis of five *E. anophelis* strains

**Figure A3:**
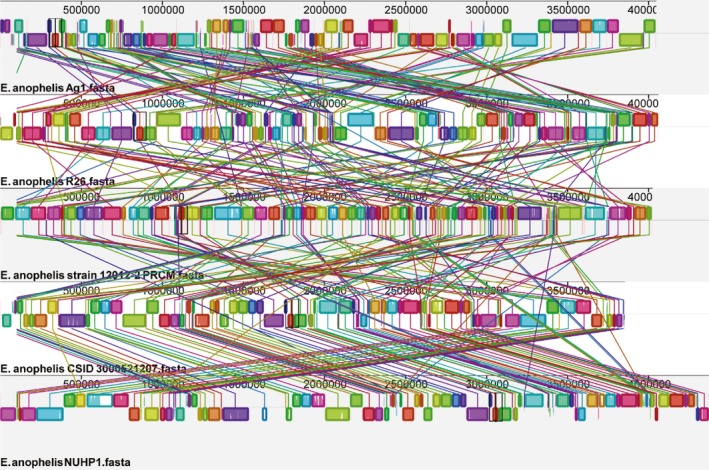
Alignment of *Elizabethkingia anophelis*12012‐2PRCM, NUHP1, CSID3000521207, Ag1 andR26 withthe progressive MAUVEsoftware. Colored blocks: a region of the genome sequence which was assumed to be homologous and internally free from genomic rearrangement. Regions outside blocks: no homology among these genomes. Completely white areas: not aligned and possibly containing specific sequence elements to a certain genome

The selected *E. anophelis* genomes had some chromosomal rearrangements with some inversions (Figure A3) and syntenic rearrangements. However, the genome arrangement of the three clinical isolates mimicked each other. Instead, the clinical and environmental isolates showed less similarity (Figure A3).

### 3.8 CRISPR prediction in *E. anophelis *strains

Our analysis revealed that only *E. anophelis* FMS‐007 contained one complete CRISPR (GTTATATCACAAAGATATCCAAAATTGAAAGC). The other selected genomes had no CRISPR. The defense of the invasions of foreign genetic elements such as plasmids, transposons, or phages may require both restriction modification systems (RMs) and CRISPRs in *Elizabethkingia*. However, the detailed mechanisms need to be further investigated.

## 4 CONCLUSION

Genomic analysis provided partial insight on the antibiotic resistance and pathogenicity mechanisms of clinical multidrug‐resistant *E. anophelis* isolates. This could prove useful information in the development of future therapeutic regimens to eliminate the infections caused by the emerging pathogen *E. anophelis*.

## CONFLICT OF INTEREST

The authors declare that they have no conflict of interest.

## AUTHORS CONTRIBUTION

Conception and design of the work: MW, SH, and SC. Data collection: MW, HG, NL, DM, and SH. Data analysis and interpretation, manuscript writing, and critical revision of the article: all authors. Revision and editing: EDW and SC. Approval of the final version of the article: all authors.

## ETHICS STATEMENT

A informed consent was obtained from the patient's relatives to retrieve and analyze this bacterial isolate. The research was approved by the Ethics Committee of Quanzhou First Hospital.

## Data Availability

All genomic data are available through the NCBI (https://www.ncbi.nlm.nih.gov) using the corresponding accession numbers provided.

## APPENDIX 1

**Table A1:** Antimicrobial resistance profile of*Elizabethkingia anophelis *12012‐2PRCM

Antibiotic class	Antimicrobial	MIC (µg/ml)	SIR
Aminoglycoside	Amikacin	>32	R
	Gentamicin	>8	R
*β*‐lactam	Imipenem	>8	R
	Meropenem	>8	R
	Cefazolin	>16	R
	Ceftazidime	>16	R
	Cefotaxime	>32	R
	Cefepime	>16	R
	Aztreonam	>16	R
	Ampicillin	>16	R
	Piperacillin	>64	R
	Amoxicillin/Clavulanate	>16/8	R
	Ampicillin/Sulbactam	>16/8	R
	Piperacillin/Tazobactam	>64/4	R
Polypeptide	Colistin	>2	R
Sulfonamide	Trimethoprim/Sulfamethoxazole	>2/38	R
Chloramphenicol	Chloramphenicol	>16	R
Quinolone	Ciprofloxacin	>2	R
	Levofloxacin	>8	R
	Moxifloxacin	>4	R
Tetracycline	Tetracycline	>8	R

Note

MIC: minimum inhibitory concentration; SIR: sensitive (S), intermediately sensitive (IS), resistant (R)
